# Steady State and Dynamic Response of Voltage-Operated Membrane Gates

**DOI:** 10.3390/membranes9030034

**Published:** 2019-03-02

**Authors:** David Nicolas Østedgaard-Munck, Jacopo Catalano, Anders Bentien

**Affiliations:** Department of Engineering, Aarhus University, Hangoevej 2, 8200 Aarhus N, Denmark; DNMunck@eng.au.dk

**Keywords:** electrokinetic phenomena, dynamic response, membrane-based gate

## Abstract

An electrochemical flow cell with Nafion 212, aqueous LiI/I${}_{2}$ redox solution, and carbon paper electrode was operated as an electro-osmotic gate based on the Electrokinetic Energy Conversion (EKEC) principle. The gate was operated in different modes. (*i*) In normal DC pump operation it is shown to follow the predictions from the phenomenological transport equations. (*ii*) Furthermore, it was also demonstrated to operate as a normally open, voltage-gated valve for microfluidic purposes. For both pump and valve operations low energy requirements (mW range) were estimated for precise control of small flows (μL range). (*iii*) Finally, the dynamic response of the pump was investigated by using alternating currents at a range of different frequencies.

## 1. Introduction

Transport of solutions and ions in pores and membranes are of interest in many processes in biology [1,2,3,4] and synthetic materials. In the latter, attention has recently focused on developing functional nanoscopic materials with tuneable properties; e.g., in carbon nanotubes where monitoring of mass transport [5,6] and controllable, reversible mass transport has been shown [7]. A special case of interest is when the mass transport of electrolyte solutions (in charged or ionizable) membranes and nanopores is driven by an electrical potential field. This is commonly referred to as electro-osmotic flow (EOF) which enables e.g., control of flux through polymer pores by gate response to pH or voltage changes. This is of interest in DNA systems for use in bio-sensing, drug delivery, ionic circuit construction [8,9], and EOF effects have also been suggested for the field of microfluidics [10] where alternative methods for pumping are sought.

**Figure 1:**
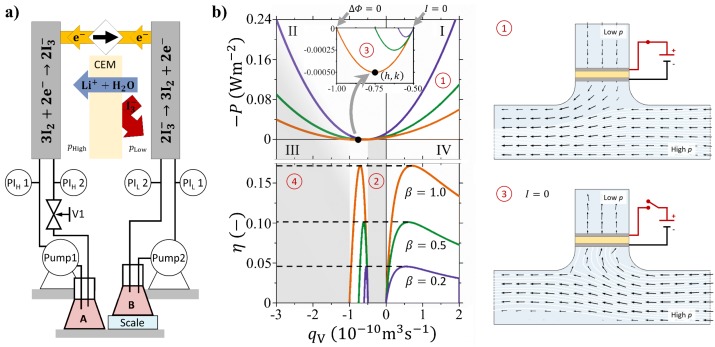
(**a**) Illustration of Electrokinetic Energy Conversion (EKEC) in a flow cell with two pumps circulating identical LiI/I_2_ solutions from reservoirs through each half cell. The illustration depicts pump operation by current induced mass transport (electro-osmosis) towards the left-hand side of the system against a pressure load. Inlet and outlet pressures in each half cell are monitored and a needle valve is used to control the hydrostatic pressure difference (Δp) across the cell. On the opposite side of the system the reservoir is placed on a scale monitoring the mass transport through the cation exchange membrane (CEM). Charge balance between the two half cells is retained by opposite redox reactions in each half cell and the net effect is migration of Li^+^ ions coupled to H_2_O-molecules through the CEM. (**b**) Illustration of the power density versus the volume flow ($q_{V}$) through the CEM. The calculations have been performed with realistic membrane transport coefficients (see main text) and with Δp=1 bar. The system is operated as a pump for P<0 and $q_{V}>0$ (quadrant I), while power generation is for P>0 and $q_{V}<0$ (quadrant III, also shown magnified in the inset).

The principles behind EOF are the rather complex electrokinetic interactions between mobile ions, polar H_2_O molecules, and immobilised charges within a charged pore or a membrane [11]. The concept of electrokinetic membrane pumping against a pressure load is illustrated in Figure 1a. A hydrostatic pressure difference (Δp) is present across a cation exchange membrane (CEM), e.g., Nafion, which separates two solutions of the same electrolyte and ionic strength. The pressure gradient induces a mass transport (in Figure 1a from the left towards right). However, if a sufficiently large electrical current is drawn in the opposite direction, ions and their hydration shells are dragged through the CEM, and a net volumetric flow towards high pressure side takes place. When CEMs are considered, the total ion flux $j_{\mathrm{ions}}=j_{+}+j_{-}$—where $j_{+}$ and $j_{-}$ are the flux of the cations and anions, respectively—is dominated by cations due to the membrane permselectivity [12]. In the case of perfect ion-selectivity the current density (*j*) is entirely driven by the cation flux and $j=F\;\cdot \;\left(j_{+}-j_{-}\right)=F\;\cdot \;j_{+}$, where *F* is the Faraday constant, while for a real membrane a fraction of the current is dissipated transporting anions. Additionally, polar H_2_O molecules are dragged together with the ions, and it results in a mass flux through the membrane, where electrical power is converted into hydraulic pumping power against a pressure gradient. This processes known as electrokinetic energy conversion (EKEC) [13,14,15,16,17]. The EKEC phenomena are reversible, meaning that applying a trans-membrane potential difference creates a solution flow against a hydraulic load and vice versa applying a hydraulic pressure difference generates electricity as streaming current and potential. From the applied current density, the mass flow permeating through the membrane can be adjusted in either direction or to zero and in this respect formally represents a voltage-operated gate. EKEC requires ions to be present in the solution and can be utilized with concentrations as low as $10^{-4}$ M, some 100 times lower than potable water. The ion concentration that maximizes the conversion efficiency is to a large extent determined by the pore size and Debye screening length of the ions in the solution. That is, for pores with larger diameter a lower concentration is needed to optimize the EKEC conversion efficiency [18].

EKEC was originally studied theoretically in the 1960’s for micro channels [19,20,21,22], and theoretical works have focused on EKEC power generation in nanochannel and nanotubes where high EKEC efficiencies (η> 50%) have been predicted due to ion-layering or slip at the pore-wall/solution interface [23,24]. High-power EKEC has been measured for glass microchannels [25,26,27,28] and we have reported that high EKEC maximum thermodynamic efficiencies (η∼ 46%) can be achieved with CEMs due to their highly charged and interconnected nanoscopic ion-channel network [29,30,31,32]. More recently, we developed an optimized electrochemical cell for the measurement of EKEC generation efficiency directly [33].

In this work we present direct measurements of the power consumption for a voltage-gate functioning with lithium iodide/iodine and a membrane electrode assembly (MEA) based on Nafion 212 and porous carbon paper. Furthermore, we experimentally investigate the dynamic response of the electro-osmotic pumping capabilities, which to the best of our knowledge has not yet been presented in literature.

## 2. Electrokinetic Pump and Valve Operation: Functioning Principle

A theoretical model to calculate the power consumption/generation for both EKEC pumping and generation can be established within the framework of the phenomenological transport equations [17]. A typical curve of the electrical power (*P*) versus the volumetric flow ($q_{V}$) is shown in the top panel of Figure 1b. Here the direction of $q_{V}$ is defined negative for a pressure-driven flow, likewise in Figure 1a the pressure difference $\Delta p=p_{\mathrm{high}}-p_{\mathrm{low}}>0$ induces a volumetric flow from the left-hand to right-hand side. In other words, $q_{V}$ is considered positive when the system is operated as a pump and the current circulates from the left-hand to the right-hand electrodes in Figure 1a.

An isothermal system close to equilibrium, with negligible concentration difference between the two reservoirs, can be fully described by a linear combination between two fluxes (current density, *j*, and volumetric flow $q_{V}$) and two driving forces (hydrostatic pressure difference, Δp and electrical potential difference, Δϕ) [18,21,22,34]. In the context of the present work, the electrical work and hydraulic work ($P_{\mathrm{hyd}}$) are defined per unit of membrane area (*A*), whereas in Ref. [17] they were derived as volumetric properties, and are as follows:

(1) P=j·Δϕ

(2) $$P_{\mathrm{hyd}}=q_{V}\;\cdot \;\Delta p$$

where j=I/A is the current density. *P* assumes negative values when power is provided to the system; that is when the system is operated as a pump ($q_{V}>0$, region 1 in Figure 1b) or when the direction of the electro-osmotic flow is the same as the pressure driven flow (region 4 in Figure 1b). At the line $q_{V}=0$ the pressure-driven flow and the electro-osmosis are exactly counterbalanced and the system is operated as a closed gate (boundary between region (1) and (2)). The quadrants I-IV are defined by the signs of $q_{V}$ and *P*. Quadrant III ($q_{V}<0$ and P>0) describes the system when it is operated as a generator and the kinetic energy of a trans-membrane pressure difference is converted into electrical power as shown in the inset of the top panel of Figure 1b. It is noted that the power generated from EKEC is several orders of magnitude smaller than the typical power either for pumping or for valve operations.

The efficiencies (η) for pump operation ($\eta =-P/P_{\mathrm{hyd}}$) and EKEC conversion ($\eta =-P_{\mathrm{hyd}}/P$) are reported in the bottom panel of Figure 1b. It is only in quadrants I and III, where the signs of $P_{\mathrm{hyd}}$ and *P* are opposite, that η is well-defined. The maximum efficiency ($\eta_{\max}$), which is the same value both for pumping and generation, can be defined by the compact notation introduced by Morrison and Osterle [21]: $\eta_{\max}=\left(\sqrt{\beta +1}-1\right)/\left(\sqrt{\beta +1}+1\right)$ where β is the EKEC figure-of-merit. β can be expressed in terms of observable transport properties and we use $\beta =\nu^{2}\sigma /\kappa_{H}$, where $\kappa_{H}$ is the hydraulic permeability, σ is the ion conductivity, and ν is the streaming potential coefficient [18,35].

The electrical power can be expressed as a function of $q_{V}$ through the equation:

(3) $$P=a\;\cdot \;{\left(q_{V}-h\right)}^{2}+k$$

The derivation of Equation (3) is straightforward from Ref. [17] and the parameters are: $a=-\Delta x/\left(\beta \kappa_{H}A^{2}\right)$, $h=-\kappa_{H}\left(1+\frac{\beta }{2}\right)A\left(\Delta p/\Delta x\right)$, and $k=\frac{\beta }{4}\Delta x\kappa_{H}{\left(\Delta p/\Delta x\right)}^{2}$, and Δx is the membrane thickness. The point $\left(h,k\right)$ represents the conditions (flow and power, respectively) of maximum power generation. The top panel of Figure 1b plots the electrical power density for three different realistic values of the figure-of-merit: β=0.2, 0.5, and 1.0, corresponding to η∼5%, 10%, and 17%. The remaining parameters used are: $\kappa_{H}=1\times 10^{-17}$ m${}^{2}$ Pa${}^{-1}$ s${}^{-1}$, $A=2.5\times 10^{-3}$ m${}^{2}$, $\Delta x=1\times 10^{-4}$ m, and $\Delta p=10^{5}$ Pa. The values of the parameters have been chosen to be representative of the properties of Nafion 212 membranes [33,36] and of the experimental conditions described in the present work. From the analysis of Equation (3) it is clear that, besides the membrane geometry and trans-membrane pressure difference, the electrical power depends only on the hydraulic permeability and the efficiency of the process which is conveniently expressed by β.

Finally, the intercept of *P* with the y-axis is the condition of the closed-gate and the power consumption is described as:

(4) $${\left.P\right|}_{q_{V}=0}=-\kappa_{H}{\left(\frac{\Delta p}{\Delta x}\right)}^{2}\left(\frac{1}{\beta }+1\right)\Delta x$$

Equations (3) and (4) are valid when the only contribution to the (electrical) resistance of the system comes from the membrane ($R_{\mathrm{cell}}=R_{\mathrm{mem}}=\frac{\Delta x}{\sigma A}$). When a real system is considered and additional parasitic resistances are present e.g., electrode charge transfer resistance, Equations (3) and (4) are still valid but β is replaced with an apparent value: $\beta_{\mathrm{app}}=\frac{\nu^{2}\sigma_{\mathrm{app}}}{\kappa_{H}}$, where $\sigma_{\mathrm{app}}$ is the apparent conductivity calculated from the total cell resistance.

## 3. Materials and Methods

### 3.1. Chemicals

The LiI/I_2_ solutions were prepared using Lithium Iodide, LiI (Sigma-Aldrich: Lithium Iodide Hydrate, 223816, St. Louis, MO, USA), in Milli-Q Water and were saturated with Iodine, I_2_ (Sigma-Aldrich: Iodine, 229695). Nafion 212 (Nafion Store: N212, ion exchange capacity 0.95 to 1.01 meq g${}^{-1}$ and thickness of 50 μm in dry conditions) was pre-treated as described earlier [36]. First it was boiled in 3 wt% H_2_O_2_ for 1 h, washed in boiling in milli-Q water for 10 min, boiled in 0.05 M sulfuric acid for 30 min, and finally rinsed several times in boiling water. After pre-treatment the membranes were stored in 1 M LiCl until use.

### 3.2. Electrochemical Flow-Cell

The flow-cell (see also Ref. [36] for additional details) is a sandwich composed of several components: stainless-steel end plates (EP), Viton and Teflon insulators (In), gold plated copper current collectors (CC), graphite blocks with machined interdigitated flow patterns (GB), 0.1 mm porous carbon paper electrodes (Fuel Cell Store: Toray Carbon Paper pre-treated in air at 500 ${}^{{}^{\circ}}$C for 7 h) (E), and a Nafion 212 Membrane (M). These components were assembled in the following configuration: EP–In–CC–GB–E–M–E–GB–CC–In–EP. The interdigitated flow fields were lowered 0.1 mm into the graphite blocks and contained each 12 channels (depth = width = 1 mm) parted by a 2 mm wide flow wall. Around the flow pattern an outer and inner Viton O-ring (M Seals) were placed. The cell was tightened with a torque of ∼4 Nm. Fittings, tubing and valves were made in PFA (Swagelok). On each side of the cell, pumps (P1 and P2, Cole-Parmer: HV-07554-85, HV77250-62) were circulating LiI/I_2_ solutions from reservoirs A and B to minimize concentration polarization, while monitoring inlet and outlet pressures (PI, Druck DE, range 1–10 bar absolute). On the high-pressure (left-hand) side, a needle valve was installed on the outlet tube, thereby enabling control of the differential pressure difference between the two sides of the cell. As the flow cell was operated in counter-flow conditions, the pressure difference was calculated as the logarithmic mean: $\Delta p_{\mathrm{LM}}=\left(\Delta p_{1}-\Delta p_{2}\right)/\left(\ln\Delta p_{1}-\ln\Delta p_{2}\right)$ with $\Delta p_{1}$ being the pressure difference between inlet of the high-pressure chamber with respect to the outlet of the low-pressure chamber and vice versa for $\Delta p_{2}$. To simplify the notation, in the following the subscript “LM” will no longer be explicitly stated. The reservoir connected to the right-hand side of Figure 1a was placed on a scale (Sartorius Quintix224, resolution 0.1 mg) to monitor the mass permeated through the membrane over time.

Before performing the experiment, the redox solution was flushed through the flow cell from the same reservoir for approximately 30 min corresponding to a circulated volume several orders of magnitude higher than the volume of the flow patterns. During this time, the electrical potential difference between the two sides of the cell was monitored and gave an indication of electrolyte concentration differences between the two sides. The system was considered stable when the potential difference between the two half-cells was lower than 20 μV. From the Nernst equation, the concentration ratio between the two half-cells could be estimated to be lower than 1.0008 and negligible in the context of the present work.

During experiments the combined reservoir was split into two galvanic isolated reservoirs. Depending on the experiment, a pressure gradient and/or an electrical current was applied across the membrane. At fixed trans-membrane pressures differences (Δp=0, 1, and 2 bar) polarization curves were performed by increasing the applied current in steps ∼2 mA every 10 s, in the range $\left[-94,105\right]$ mA. The characteristic pump curves were done by holding the applied current (−42.3 mA for 25 cm${}^{2}$) and changing the pressure gradient in steps covering the entire pump curve which ranges between no pressure difference (maximum flow) and zero mass transport through the membrane (maximum pump head). Valve operation was engaged by applying an electrical current that precisely counter-balanced the applied pressure difference and resulted in zero mass transport. These experiments have been performed at room temperature (21±1 ${}^{{}^{\circ}}$C) and the data recorded at 0.5 Hz.

For stability and noise suppression the measurements of the permeated flow related to the dynamic response of the mass transport were conducted without the circulation pumps running. Sinewave current with fixed amplitude and with varying frequencies were supplied from a programmable source/load (Agilent U2722A) controlled with NI LABview software. Depending on experimental duration, several cycles were run for each frequency. Additionally, four-point Electrochemical Impedance Spectroscopy (EIS) (CH Instruments, CHI660E) was performed to analyse the electrical response between 10${}^{-4}$ Hz to 10${}^{5}$ Hz (12 points per decade). The EIS was also used as an AC source, with fixed amplitude of the electrical potential difference, to measure the phase shift and amplitude of the electro-osmotic flow with respect to the electrical current flowing through the membrane.

## 4. Results and Discussion

### 4.1. Pump and Valve Operation

**Figure 2:**
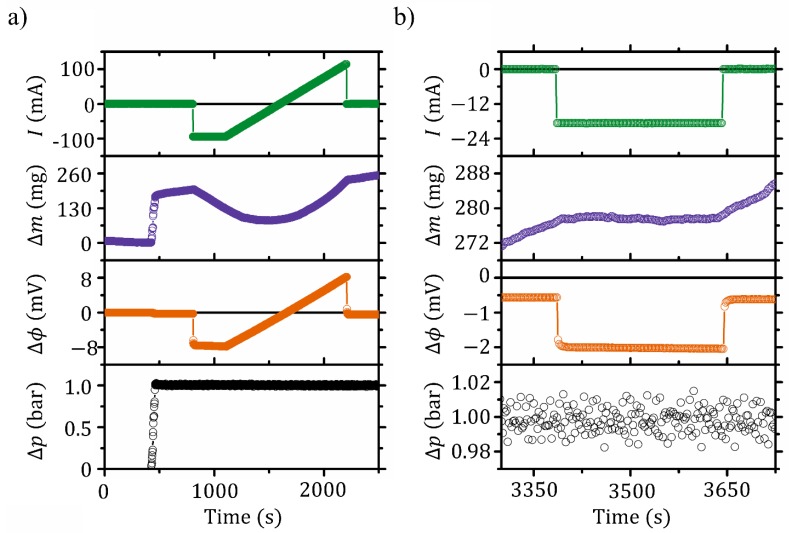
Raw data for the electrical current (*I*), mass transport (Δm), potential difference (Δϕ), and logarithmic mean pressure difference (Δp) across the membrane as function of time. Noise levels are less than: 0.05 mA, 0.5 mg, 0.2 mV, and 0.04 bar, respectively. All values are positive toward the left-hand with respect to Figure 1a. (**a**) Shows raw data of an entire polarisation experiment; (**b**) is an excerpt of an event where the EKEC valve is closed and then opened again.

Figure 2a displays an example of raw data for an entire polarization curve as function of time; from top to bottom: current (*I*) entering the electrochemical flow cell, mass change (Δm) in the right-hand side reservoir (see Figure 1), potential difference (Δϕ) between the two sides of the cell, and pressure difference (Δp) across the membrane. At time t∼500 s, Δp∼1 bar is applied. A response is seen in the left reservoir as an instantaneous increase in Δm∼200 mg, due to the mechanical deformations of the membrane followed by a pressure-driven mass transport seen as a linear mass increase. This is accompanied by an instantaneous change in the Δϕ∼0.3 mV that can be observed as a small step change in the curve around t∼500 s. The experiment at this point is conducted open circuited (I=0). In this condition the streaming potential coefficient ($\nu ={\left.\Delta \phi /\Delta p\right|}_{I=0}$) can be calculated corresponding to 2.8 nV Pa${}^{-1}$ for LiI/I_2_ solutions at 1 M Li^+^ and in good agreement with previous work [33]. The volumetric flow rate ($q_{V}=\frac{dm}{dt}/\tilde{\rho }$) through the membrane is found as the derivative of the mass vs. time of sets of 30 data points (60 s). Here we assume the density of the solution permeated to be that of pure water, i.e., $\tilde{\rho }$ is the density of water at 300 K. At I=0, $\kappa_{H}$ can be found from $q_{V}=\kappa_{H}A\;\cdot \;{\left.\frac{\Delta p}{\Delta }\right|}_{I=0}$. To account for the electroviscous effects, an intrinsic hydraulic permeability coefficient ($\kappa_{H}^{⋆}$) can be defined [17]. This can be found in the same manner with $q_{V}=\kappa_{H}^{⋆}A\;\cdot \;{\left.\frac{\Delta p}{\Delta }\right|}_{\Delta \phi =0}$, however, in this case it is measured under closed circuited (Δϕ=0) conditions. For Nafion 212 with LiI/I_2_ solution at 1 M Li^+^ we measured $\kappa_{H}=2.0\times 10^{-17}$ and $\kappa_{H}^{⋆}=2.3\times 10^{-17}$ m${}^{2}$ Pa${}^{-1}$ s${}^{-1}$.

From about t∼800 s to ∼2300 s a polarization curve is recorded, and the current is swept from around −100 mA to 100 mA. This is seen as the parabolic curve of the raw mass data. With I∼20 mA a balance between the electro-osmotic flow and the pressure-driven flow is obtained and results in $q_{V}=0$.

Figure 2b reports a magnification on a few hundred seconds where the membrane-based gate is operated as a valve. Before t=3400 s the hydraulic permeability is clearly seen as a linear mass increase. At t∼3400 s the mass transport is ceased when I∼19 mA is delivered to the cell and corresponds to closing the valve. The current required to close the valve is within 5% and in agreement with the value obtained during the polarization curve. Also a relatively short actuation time is observed while turning off the valve. It is anticipated that both the slight higher current required to operate the valve with respect to the polarization curve and the delay time are related to the time lag of the mass transport which is discussed below. At time t∼3650 s the valve is again opened by setting the current to zero.

**Figure 3:**
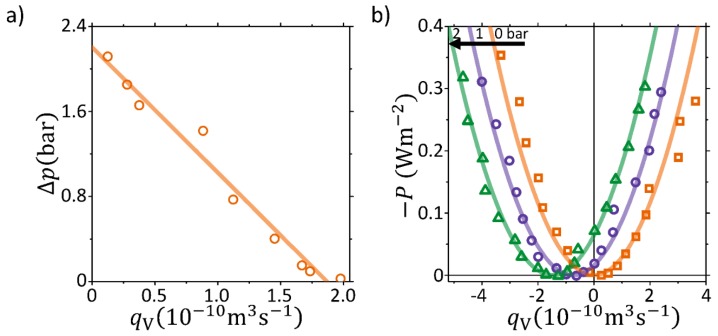
(**a**) Pump curve at I=−42.3 mA, pressure difference (Δp) versus volume flow ($q_{V}$) through the membrane, for the EKEC microfluidic pump. (**b**) Electrical power (*P*) versus $q_{V}$ for 0, 1, and 2 bar pressure difference. Lines are fits to the model of Equation (3), yielding $\kappa_{H}=1.55\times 10^{-17}$ m${}^{2}$ Pa${}^{-1}$ s${}^{-1}$ and β=0.21 for A=25 cm${}^{2}$ and membrane thickness (in fully hydrated conditions) Δx=60μm. Each data point is calculated by an average of 30 raw data points in a 60 s time series.

Figure 3a shows the pump curve obtained at I=−42.3 mA where a maximum pump head of ∼2.2 bar and a maximum volumetric flow of $q_{V}∼1.9\times 10^{-10}$ m${}^{3}$ s${}^{-1}$ is obtained. The pump curve is fitted to [17]:

(5) $$\Delta p=-\frac{\Delta x}{\kappa_{H}A}\;\cdot \;q_{V}-\frac{\nu \Delta x}{\kappa_{HA}}\;\cdot \;I$$

and yields values of $\kappa_{H}=2.0\times 10^{-17}$ m${}^{2}$ Pa${}^{-1}$ s${}^{-1}$ and $\nu =-4.4\times 10^{-9}$ V Pa${}^{-1}$. Here we note the remarkable match between the present calculated value of the hydraulic permeability with respect to that measured from Figure 2 ($\kappa_{H}=2.0\times 10^{-17}$ m${}^{2}$ Pa${}^{-1}$ s${}^{-1}$). This suggests that the above method can be used as an alternative approach to quantify $\kappa_{H}$. On the other hand, the streaming potential coefficient seems overestimated (around 50%).

In Figure 3b the raw data of Figure 2a are analysed and it shows the electrical power versus $q_{V}$. Besides the experiment with Δp=1 bar (blue circles) it also includes experiments with Δp=0 and 2 bar, shown as red squares and green triangles, respectively. An expected shift towards more negative values of the flow is observed for increasing Δp. These parabolic curves can be described by Equation (3) and the fits are shown as solid curves in Figure 3b, where $\kappa_{H}=1.55\times 10^{-17}$ m${}^{2}$ Pa${}^{-1}$ s${}^{-1}$ and $\beta_{\mathrm{app}}=0.21$ are found for Nafion 212 membrane with A=25 cm${}^{2}$ and Δx=60 μm. Here we note that value of $\kappa_{H}$ is comparable (around 25% lower) with respect to that calculated at zero current density. Additionally, the calculated $\beta_{\mathrm{app}}$ is the apparent one, which is diminished with respect to the intrinsic membrane β. From the measured electrical resistance from EIS of the membrane (0.015 Ω) and the total cell (0.07 Ω)) an intrinsic η∼19% can be calculated. The value of β is comparable with respect to that of Nafion 117 where an apparent efficiency was previously measured to η∼19% with its corresponding intrinsic η∼25% [33]. On the other hand, $\beta_{\mathrm{app}}$ seems systematically lower: this is expected and it is a result of the larger relative influence of the parasitic resistances in the much thinner Nafion 212 with respect to Nafion 117 membrane.

By using the transport coefficients obtained for Nafion 212 the power consumption in different conditions can be calculated. If for instance an active area of 1 cm${}^{2}$ is considered and hydraulic loads of 1, 10, and 100 bar are applied, it would require 0.004 mW, 0.4 mW, and 40 mW to enable valve operation, respectively. Considering the same hydraulic loads, in pump mode, a flow of 1 μLs${}^{-1}$ would require 0.62 W, 0.65 W, and 1.10 W, respectively. These calculations are conservative since they consider the real cell resistance obtained in the present work, hence lower power densities are required for more optimized systems.

As a *proof-of-concept* we show a video in the Supplementary Information of how a microfluidic EKEC pump can control the EKEC pumping through a cell connected to a microchannel.

### 4.2. Dynamic Response

To investigate in more detail the dynamic response of the voltage-operated gate a series of alternating current experiments were conducted. A similar technique was recently used for studying the fouling on ultrafiltration membranes [37]. The streaming current developed from sinusoidally alternating fluid flow forced through a cylindrical capillary was also investigated in Ref. [38]. However, in this latter work no mention of any time lag has been reported, and this might be due to the large dimension of the capillary (0.196 cm ID).

**Figure 4:**
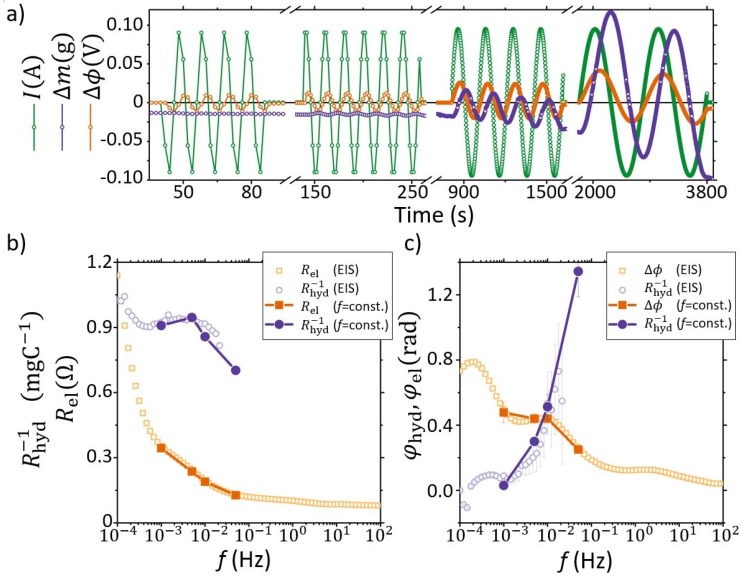
(**a**) Raw data for the dynamic response with Nafion 212. An alternating current (*I*, green) is applied with constant frequency and amplitude. The phase shift between the potential difference (Δϕ, orange) and the mass transport through the membrane (Δm, purple) is in particular seen at low frequencies. (**b**,**c**) Bode plot of the data in panel a along with results from Electrochemical Impedance Spectroscopy (EIS) (**b**) amplitude of the sinewave for the electro-osmotic coupling factor ($R_{\mathrm{hyd}}^{-1}=\frac{\Delta m}{\Delta t}/I$, purple) and magnitude of electrical impedance ($R_{\mathrm{el}}=\Delta \phi /I$, orange) versus frequency (*f*). (**c**) Phase shift (φ) for Δϕ and Δm/Δt with respect to *I*. In the Bode plot two independent measurements are shown, one is obtained with an EIS station (hollow symbols), while the other is obtained with a programmable electrical source/load (filled symbols).

The experiments were carried out with the same cell used for the previously pump curves. However, to increase the resolution of the mass transport measurements, the circulation pumps were not running during experiments thus reducing the noises on the measurement from 15 mg down to the resolution of the scale (0.1 mg). In between experiments, the circulation pumps were used to flush the cell to ensure equal concentration in both chambers. During experiments, alternating currents were applied to the cell with a fixed amplitude of 95 mA at different frequencies (f=0.1, 0.05, 0.005, and 0.001 Hz) each for several cycles, while at the same time monitoring the potential of the cell. The raw data obtained are shown in Figure 4a where the green, orange, and purple lines represent *I*, Δϕ, and Δm, respectively. For f=0.001 Hz a clear phase shift between *I*, Δϕ, and Δm can be observed directly from the raw data.

Figure 4b is a Bode plot of the raw data from Figure 4a. In addition to the frequency dependence of the electrical impedance, Figure 4b also shows the frequency dependence of what we term the electro-osmotic (EO) coupling factor $R_{\mathrm{hyd}}^{-1}=\frac{\Delta m}{\Delta t}/I$. Here the EO coupling factor represents the mass flow transported through the membrane for a unit of electric charge (kg C${}^{-1}$). In an additional independent experiment also included in Figure 4b,c, an EIS station was used as an AC source while collecting data for the mass transport, with a constant perturbation amplitude of Δϕ=14.7 mV (see Supplementary Information for raw data). From both experiments, it is seen that $R_{\mathrm{el}}$ increases monotonously with decreasing frequencies and does not reach a plateau because the system is limited by the mass transport since the solutions were not circulated during the AC tests. Similarly, the EO coupling factor also has a steep increase with decreasing frequencies in the diffusion limited regime; however, in this case $R_{\mathrm{hyd}}^{-1}$ appears stable in the frequency range from about $3\times 10^{-4}$ Hz to about $2\times 10^{-2}$ Hz. At frequencies above $2\times 10^{-2}$ Hz, $R_{\mathrm{hyd}}^{-1}$ resumes its decreasing trend, which indicates that the EKEC efficiency decreases (rather steeply) as the frequency is increased over this value.

With respect to the frequency response of the phase shift ($\varphi_{\mathrm{hyd}}$ and $\varphi_{\mathrm{el}}$), it is seen that both $\varphi_{\mathrm{el}}$ and $\varphi_{\mathrm{hyd}}$ show relative maxima at frequencies ∼$5\times 10^{-4}$ Hz. We attribute the relative maximum of $\varphi_{\mathrm{hyd}}$ at low frequencies to the diffusion-limited mass transfer. Indeed both the peak position, which is related to the apparent diffusion coefficient of the ions in the membrane ($D_{\mathrm{ion}}^{\mathrm{app}}$), and its magnitude (order of 0.1 radians) are compatible with the predicted values from the theory of AC electro-osmotic flow in charged nanocapillary [39]. This non-monotonous behaviour is related to the ion transport through the selective membrane and the effect of the change in ion concentration (concentration polarization phenomena due to electro-osmosis) on the stagnant boundary diffusion layers at the membrane-solution interfaces. Indeed we recall that the dynamic experiments were conducted without circulating the solutions in the flow cell. In this work we found $D_{\mathrm{ion}}^{\mathrm{app}}$ two orders of magnitude lower than the diffusion coefficient values for Li^+^ or I_3_^−^ in water ($D_{\mathrm{Li}}=1.03\times 10^{-9}$ m${}^{2}$ s${}^{-1}$). This difference is expected due to the influence of the porosity and tortuosity factors of the ion-channel network and it is compatible with reported values in the literature (see e.g., Appendix in Ref. [40]). Hence the dynamic response of the fluid flow applying alternating current can be used to assess $D_{\mathrm{ion}}^{\mathrm{app}}$. To the best of our knowledge this is the first time that the diffusion coefficient in membranes has been measured via electro-osmotic flow driven by AC. This opens the possibility of using the AC method described in the present work as a starting point to develop a method for measuring the apparent diffusion coefficient or cross-over of ions in membranes. (It is emphasized that $D_{\mathrm{ion}}^{\mathrm{app}}$ is an apparent property and should be treated as an engineering parameter that enables us to calculate the ion cross-over with the knowledge of only the macroscopic geometrical properties of the membrane (i.e., membrane area and thickness). This has the main advantage of not requiring any information of the actual values of the porosity and tortuosity factor, which can be particularly challenging to assess.)

At frequencies $>9\times 10^{-2}$, $\varphi_{\mathrm{hyd}}$ appears to monotonously increase toward a π/2 shift with respect to the applied current (and in phase with the voltage difference, see Supplementary Information). Here it is noted that the uncertainty of the data collected from the scale increases significantly for higher *f*, and ultimately limited the data acquisition and analysis for the mass transport to 0.02 Hz. Nonetheless, Figure 4c indicates that at relatively high frequencies the phase shift between the current and the mass flow increases. At high frequencies, $\varphi_{\mathrm{hyd}}$ is responsible for the observed time delay during actuation of the electro-osmotic valve, which can be estimated to be at the most of the order of one second.

## 5. Conclusions

An EKEC flow cell equipped with a Nafion/carbon paper electrode assembly was operated as a voltage-operated gate. Input power, *P*, versus volumetric flow rate, $q_{V}$, was shown to follow the theoretical curve derived from the phenomenological approach: $P=a\;\cdot \;{\left(q_{V}-h\right)}^{2}+k$, with the parameters *a*, *h*, and *k* only dependent on the hydraulic permeability coefficient, $\kappa_{H}$, and the electrokinetic figure-of-merit, β. The same EKEC flow cell was demonstrated to operate as a normally-open, voltage-gated, valve. Both pump and valve operations were demonstrated for microfluidic purposes due to the small energy requirements (mW range) and precise control of small fluxes (μL range or lower). Additionally, the time-lag of the system was investigated by applying an alternating current to the flow cell system at varying frequencies. From this EKEC the pump and valve actuation response times were estimated, along with an experimental indication that the AC method could in principle be used for evaluating the apparent diffusion coefficient or cross over of ions in membranes.

## Supplementary Materials

The following are available online at https://www.mdpi.com/2077-0375/9/3/34/s1, Figure S1: Experimental data (normalized) and best fitting for the electro-osmotic flow and voltage driven by an alternating current at several frequencies, Figure S2: Normalized mass flow, electrical potential difference, and current (I), Amplitudes for the differential mass and electrical current used in the normalization reported in Figures S1 and S2, Video S1: EKpump.
