# Supplementary material for: Steady State and Dynamic Response of Voltage-Operated Membrane Gates
Source: Membranes (Basel). 2019 Mar 2;9(3):34. doi: 10.3390/membranes9030034 (PMC6468597; doi:10.3390/membranes9030034)
Supplement: Supplementary file 1 [file membranes-09-00034-s001.zip › Supplementary Information_EKEC_pump_final.docx]

*Supplementary Information for*

**Steady State and Dynamic Response of Voltage-Operated Membrane Gates**

David Nicolas Østedgaard-Munck, Jacopo Catalano^*^, and Anders Bentien^[[1]](#footnote-1)^**

Department of Engineering, Aarhus University, Hangoevej 2, 8200 Aarhus N, Denmark

Dynamic measurements of the electro-osmotic flow were performed by using an electrochemical work station as AC source in a frequency range $f=\left[ {10}^{-4},0.02 \right]$ Hz. To highlight the phase shift between the differential mass, $\Delta m$, and the voltage difference, $\Delta\phi$, Figure S1 reports the normalized values of $\Delta m$ and $\Delta\phi$ versus the dimensionless time, $t^{*}$, calculated as the product of the dimensional time and the applied AC frequency. In Figure S1 the symbols represent the raw data, after normalization, as collected from the scale and acquisition card (National Instruments), while the solid lines are the best fits of the experimental data. Generally,$\Delta m$ follows closely a sinusoidal function with exception of the data at $f=1.78 {10}^{-2}$ and 2$.15 {10}^{-2}$ where a noise in the apparatus affected the measurements. At frequencies above 2$.6 {10}^{-2}$ Hz, the number of the experimental points was too limited (since the differential mass was sampled at 0.5 Hz from the National Instruments card) and the uncertainties of the measurement were too high, and therefore the experimental data discarded.

The fits of the differential mass were afterwards used for calculating the mass flow, via differentiation, and they were coupled with the current density acquired via the electrochemical workstation. The mass flow ($d\Delta m/dt$), electrical potential difference, and current (*I*) are reported after normalization in Figure S2. It is clearly seen that the phase shift between the applied current and the mass flow is almost zero at the lowest frequencies, while is rather large ($\sim\frac{\pi}{2})$ when the frequency is increased to $\sim2 {10}^{-2}$ Hz.

The amplitudes of the differential mass and current used in the normalization reported in Figures S1 and S2 are shown in Figure S3, while the electrical potential difference was held constant at $\Delta\phi=14.7$ mV.


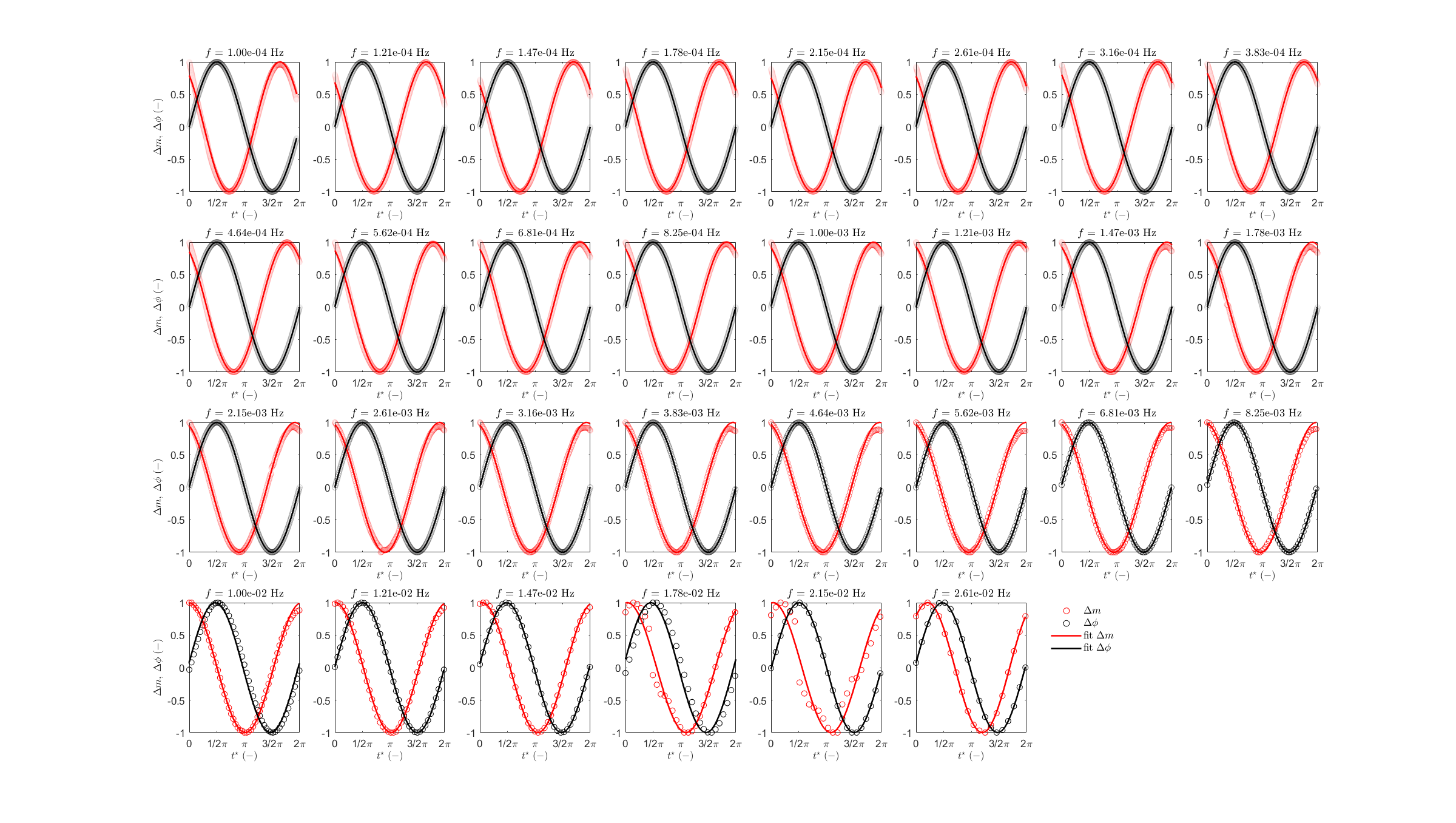


**Figure S1** Experimental data (normalized) and best fitting for the electro-osmotic flow and voltage driven by an alternating current at several frequencies.


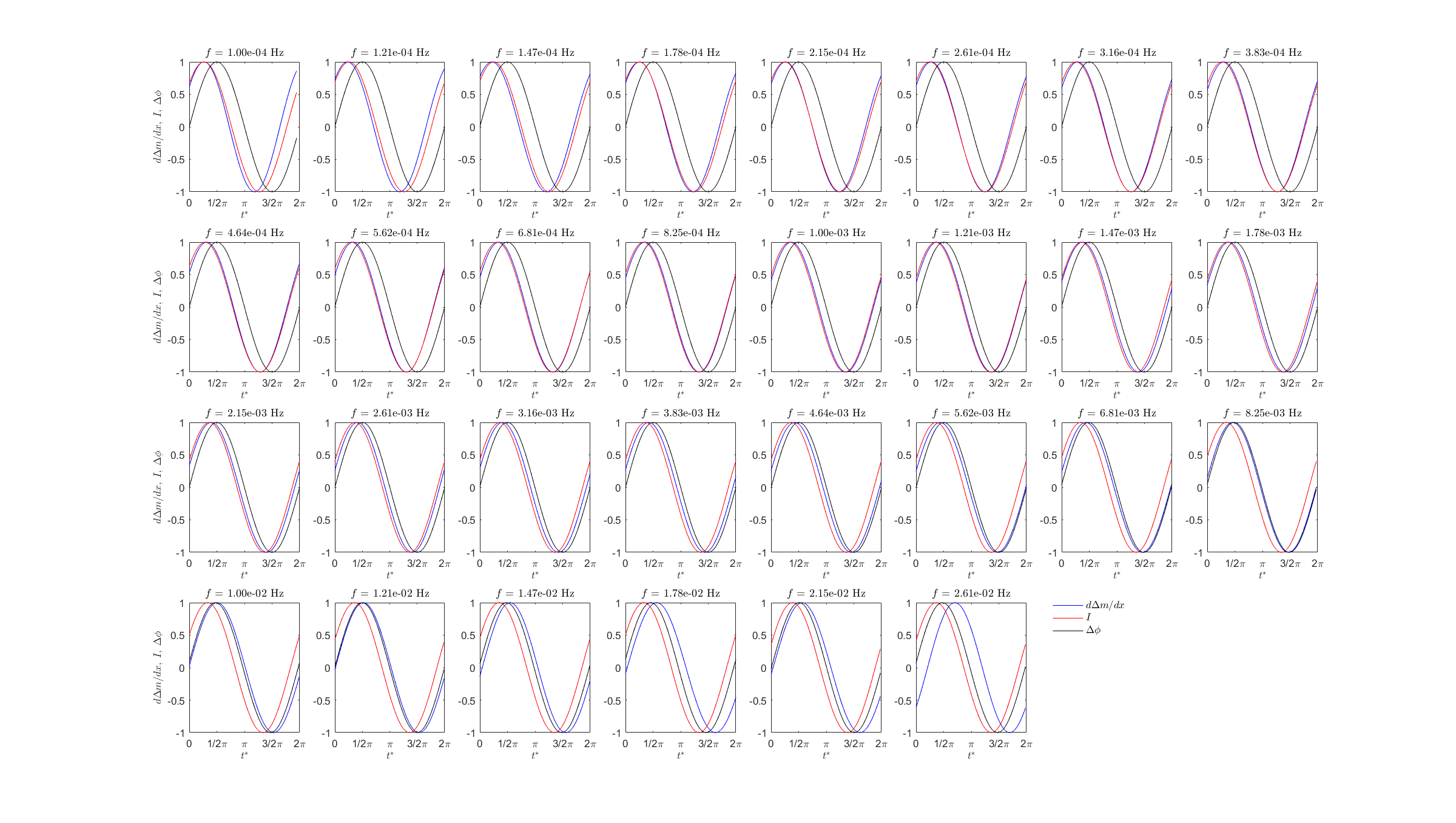


**Figure S2** Normalized mass flow ($d\Delta m/dt$), electrical potential difference, and current (*I*).

**
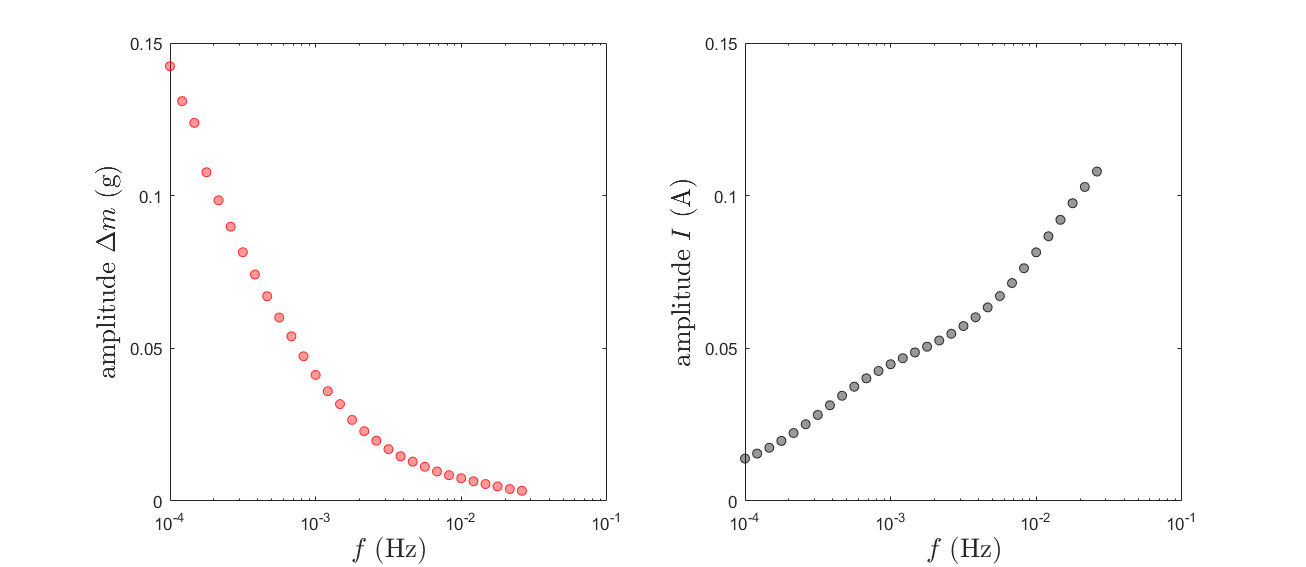
**

**Figure S3** Amplitudes for the differential mass and electrical current used in the normalization reported in Figures S1 and S2.

1. * Corresponding authors. E-mail addresses:^*^ jcatalano@eng.au.dk (J. Catalano);^**^ bentien@eng.au.dk (A. Bentien). [↑](#footnote-ref-1)
